# PrEP policy implementation gaps and opportunities in Latin America and the Caribbean: a scoping review

**DOI:** 10.1177/20499361231164030

**Published:** 2023-04-17

**Authors:** Luke Murphy, Andrea Bowra, Ellithia Adams, Robinson Cabello, Jesse L. Clark, Kelika Konda, Amaya Perez-Brumer

**Affiliations:** David Geffen School of Medicine, University of California Los Angeles, Los Angeles, CA, USA; Division of Social and Behavioural Health, Dalla Lana School of Public Health, University of Toronto, Toronto, ON, Canada; Division of Social and Behavioural Health, Dalla Lana School of Public Health, University of Toronto, Toronto, ON, Canada; Asociación Civil Vía Libre, Lima, Peru; David Geffen School of Medicine, University of California Los Angeles, Los Angeles, CA, USA; Universidad Peruana Cayetano Heredia, Lima, Peru; Department of Population and Public Health Sciences, Keck School of Medicine, University of Southern California, Los Angeles, CA, USA; Division of Social and Behavioural Health, Dalla Lana School of Public Health, University of Toronto, 155 College Street, Toronto, ON, Canada, M5T 3M7

**Keywords:** HIV prevention, implementation, Latin America, policy, PrEP

## Abstract

**Background:**

Pre-exposure prophylaxis (PrEP) is an important tool for HIV prevention in Latin America and the Caribbean (LAC). Yet, little is known about the PrEP policies landscape in the region. Addressing this gap, this scoping review assessed current PrEP policies throughout LAC to better understand existing PrEP implementation gaps and identify opportunities to improve access.

**Methods:**

We conducted a scoping review, using a modified PRISMA extension, through 28 July 2022, to identify country-level PrEP policies. Data were collected in English, Spanish, French, and Portuguese utilizing online platforms for screening and data extraction (Google Forms, Zotero, and Excel). Extracted data were divided by data source, including country-level government policies, gray literature, and peer-reviewed literature, with at least one full-text reviewer and data extractor per publication. An iterative summative content analysis was performed to compare and interpret themes across phases and data sources.

**Results:**

Of the 33 countries in LAC, 22 (67%) had policies approving daily oral PrEP for HIV prevention, which outlined specific key populations, including men who have sex with men, transgender women, sex workers, and serodiscordant couples. Generic tenofovir disoproxil fumarate/emtricitabine has been approved in 15 of the 33 countries, and 13 of the 33 countries have incorporated PrEP into their public health system. No countries were found to have approved cabotegravir. Costing data were reported by only one country, Ecuador, in its national health ministry guidelines. Findings also document a lag between the media/gray-literature announcement of PrEP and implementation of policies.

**Conclusion:**

Findings underscore significant advances in PrEP policies in the region and signal opportunities for greater PrEP implementation. Since 2017, an increasing number of countries have begun to provide PrEP to communities at heightened need, although significant gaps remain. Policy approval is a key step to further increasing access to PrEP in LAC, necessary to reduce the burden of HIV in LAC, specifically among marginalized populations.

## Introduction

Having proven effective in reducing new HIV infections in diverse settings and among those who experience a disproportionate burden of disease, such as men who have sex with men (MSM) and transgender women (TW), pre-exposure prophylaxis (PrEP) presents an important and necessary tool for HIV prevention in Latin America and the Caribbean (LAC).^1,2^ The HIV epidemic in LAC is concentrated among MSM and TW in urban areas.^3^ In many countries across the region, HIV prevalence is above 10% among MSM and often above 20% among TW.^4^ Since 2010, many countries in LAC have made significant progress toward the UNAIDS 95-95-95 testing and treatment goals, which specify 95% of all people living with HIV will know their HIV status, 95% of all people diagnosed with HIV infection will receive antiretroviral therapy (ART), and 95% of all people receiving treatment will maintain consistent viral suppression.^5^ Despite advances toward these goals, specifically in HIV testing and ART coverage, HIV prevention remains a challenge in LAC, with the incidence of new cases declining only 1% since 2010.^6^ Although treatment should reduce the number of non-virally suppressed individuals and community-level circulation of virus, prevention requires more work than solely relying on a treatment-as-prevention strategy.

PrEP was first approved in the United States in 2012 and subsequently recommended by the World Health Organization (WHO) in 2014. Since that time, global access to PrEP has been uneven, with greater availability in the Global North and parts of Sub-Saharan Africa compared with other regions.^7,8^ The United States, Canada, Australia, France, Kenya, and South Africa are among the countries with the highest number of people starting PrEP, with far fewer initiations in most of LAC.^9^ In comparison, the expansion of treatment and prevention measures in sub-Saharan Africa, which has a higher burden of disease from HIV, has led to a decrease in HIV incidence in some countries in the region while LAC has been left behind.^10^ Expanded access to and adoption of prevention interventions, including PrEP, are urgently needed to curb the HIV epidemic in LAC.^3,4^

Three PrEP regimens are currently recommended by the WHO. The first, recommended in 2014, is daily oral tenofovir disoproxil fumarate/emtricitabine (TDF-FTC).^11^ The second recommendation, released in 2019, is for Event-Driven PrEP (ED-PrEP) or 2-1-1 PrEP. This scheme, recommended only for MSM at this time, consists of taking two pills of TDF-FTC 2–24 h before sex, one pill 24 h after the first dose, and one pill 24 h after the second dose.^12^ Most recently, in 2022, the WHO recommended that long-acting injectable cabotegravir (CAB-LA) be offered as an additional prevention option for people at substantial risk of HIV infection.^13^ Descovy, or branded tenofovir alafenamide/emtricitabine (TAF/FTC), has not been adopted into WHO PrEP guidelines.^11^ Although TAF/FTC has been proven effective as oral PrEP for cisgender males in clinical trials, it remains unavailable in LAC.^10,14^ In addition to these recommended modalities, there are promising advancements in the field of HIV prevention for increasing the reach and reducing barriers to uptake of PrEP. Currently, there are ongoing clinical trials for long-acting PrEP modalities in various formulations, including a vaginal ring, an implant, a monthly oral pill, and semi-annual subcutaneous injections.^15,16^ These long-acting forms of PrEP hold great potential for decreasing barriers to medication adherence, thereby increasing the efficacy of HIV prevention,^17^ yet the poised impact may be limited by existing implementation gaps.^15^

Further hindering the uptake of PrEP across LAC, the cost at both individual and national levels is often a significant barrier, especially among vulnerable populations.^18^ Attempting to mitigate this barrier, initiatives such as the Pan American Health Organization (PAHO) Strategic Fund exist to make PrEP, and other medications, more accessible.^19^ As of 2022, 34 countries in LAC have agreements with the PAHO Strategic Fund to procure decreased cost medications, including antiretrovirals to treat HIV as well as other essential medications such as antivirals and antimalarials. Countries opt into the fund and declare which medicines they require from the agency. To ensure a low cost of medication, PAHO has brokered long-term agreements with various pharmaceutical agencies.^19,20^ Through this purchasing agreement, a 30-day supply of generic TDF-FTC costs $4.50.^21^

Existing scholarship on PrEP in LAC argues strongly for the incorporation of PrEP into HIV prevention strategies.^3^ Recent literature has also underscored the need for targeted PrEP interventions tailored to the specific needs of the populations who experience disproportionate risks for HIV, specifically MSM and TW.^22–24^ Currently, there is an absence of any studies characterizing and comparing PrEP access and implementation across countries in LAC. Addressing this gap, this scoping review assesses the status of PrEP policies throughout LAC to better understand existing PrEP implementation gaps and opportunities to improve access. To do so, we provide an overview of the state of approval of PrEP, both branded Truvada and generic TDF-FTC, in LAC countries and describe and compare PrEP prescribing guidelines and methods of delivery across LAC.

## Methods

In accordance with the research objectives, we conducted a two-phased scoping review to examine: (a) country-level PrEP policies and (b) access across LAC. The Preferred Reporting Items for Systematic reviews and Meta-Analyses (PRISMA) Extension for Scoping Reviews^25,26^ guided our review, yet modifications were made to accommodate the dearth of literature published on PrEP policies in progress throughout the region. Specifically, targeted searches were conducted by country if initial assessment reported that no country-level policy was approved to assess regional approvals and advances made toward policy approvals. The two phases of searching, screening, and eligibility assessment are described below. Review sources consisting of policy documents, health authorities’ guidelines, media stories, organizational reports, press releases, web pages, NGO reports, personal communications, peer-reviewed articles, and other directives were published on or before 28 July 2022. The search strategies are detailed by phase below.

### Phase 1

Phase 1 corresponds to Objective 1 to examine the state of PrEP approval in LAC countries, specifically whether TDF-FTC is approved for HIV prevention. This information was obtained for each country from the country’s medication registry, country-specific guidelines for HIV/AIDS treatment and prevention, PrEP Watch, gray literature, and media articles. To begin, each country’s Ministry of Health webpage was located and searched using the navigation menus provided or using the search function with the following search terms: ‘Truvada’ OR ‘TDF-FTC’ OR ‘tenofovir’ OR ‘tenofovir disoproxil fumarate’ OR ‘emtricitabine’. If approval information was not found, country-specific guidelines were identified using the search terms ‘*guía*’ OR ‘*guía clínica*’ OR ‘*lineamientos*’ OR ‘*recomendaciones*’ OR ‘*estrategía nacional*’ AND ‘*VIH/SIDA*’. For English-speaking countries, the terms included ‘guide’ OR ‘clinical guide’ OR ‘guidelines’ OR ‘recommendations’ OR ‘national strategy’ AND ‘HIV/AIDS’. If country-specific guidelines were not available or did not contain approval information, Google search engine was explored using the same search terms AND [country name]. In addition, we searched PAHO, WHO, and other multilateral organizational websites using search terms ‘PrEP’ OR ‘pre-exposure prophylaxis’ OR ‘HIV’ OR ‘VIH’ to find information on PrEP policy implementation and development.

### Phase 2

The second phase of this review aimed to describe and compare the methods of availability of PrEP across LAC countries. This information was sourced from country-specific guidelines, government documents, press releases, and media articles. First, sources identified in Phase 1 were searched for information relevant to mechanisms of availability or prescribing guidelines. If information was not found, additional searches were conducted in Google using the search terms either in English, Spanish, or Portuguese depending on the country’s official language: [country name] AND ‘PrEP’ OR ‘Truvada’ OR ‘FTC-TDF’ OR ‘TDF-FTC’ OR ‘FTC/TDF’ OR ‘TDF-FTC’ OR ‘tenofovir’ OR ‘tenofovir disoproxil fumarate’ OR ‘emtricitabine’ AND ‘access (*acceso*)’ OR ‘public policy (*política pública*)’ OR ‘demonstration project (*proyecto de demostración*)’ OR ‘implementation project (*proyecto de implementación*)’ OR ‘pilot project (*proyecto piloto*)’ OR ‘pilot program (*programa piloto*)’.

Data were collected in English, Spanish, French, and Portuguese through a variety of online platforms for screening and data extraction (Google Forms, Zotero, and Excel). Extracted data were divided by data source, including country-specific guidelines, government documents, press releases, and media articles, with at least one full-text reviewer and data extractor per publication. Inclusion was limited to documents that addressed the approval of or access to PrEP for HIV prevention, methods of its availability, clinical guidelines, or medication cost for LAC countries. Data analysis was guided by an iterative summative content analysis meaning that initial research aims were informed by existing content, extracted content was counted and compared, and the underlying contexts of the data were interpreted.^27^ Members of the research team coded sections of the documents into overarching interpretative themes and subthemes related to how PrEP availability and access was explicitly described or implied in the sources. Codes were used to generate key themes across extracted documents and yielded summaries relevant to law, policy, and access for PrEP. Emergent themes were discussed across the full team who met weekly throughout the extraction and analysis processes to review to compare, discuss, and interpret data.

## Results

### PrEP approval status and ongoing research in the LAC region

Of the 33 countries in LAC, 22 (67%) have policies approving daily oral PrEP for HIV prevention. All 22 countries approved branded Truvada for PrEP use, and 15 (45%) had registered generic TDF-FTC for importation and sale. (Refer to Table 1 for PrEP approvals for brand name Truvada and generic TDF-FTC by country.)

**Table 1.** PrEP policies in Latin America and the Caribbean through 30 June 2022.

Country	PrEP approval	Branded Truvada approved	Generic TDF-FTC approved	Country-level PrEP guidelines	PrEP available as a public policy	Modes of PrEP availability
South America						
Argentina	Yes^28^	Yes^29^	Yes^29^	1. MSM and TW who report inconsistent condom use during anal sex, and/or diagnosis of a bacterial STI in the last 6 months, and/or use of PEP more than once. 2. Serodiscordant couples in which the HIV-positive partner does not maintain an undetectable viral load and reports inconsistent condom use during sex. 3. Sex workers with inconsistent condom use. 4. PWID who have shared needles in the last 6 months.^30^	Yes^10^	Available at the national level^9^
Bolivia	–	Yes^31^	No^31^	–	–	
Brazil	Yes^32^	Yes^33^	Yes^33^	For MSM, transgender people, sex workers: condomless anal or vaginal sex in the last 6 months, and/or recurring STIs, and/or repeated use of PEP. For serodiscordant couples: condomless anal or vaginal sex with a person who has HIV^32^	Yes^33^	Available at the national level through the public health system since 2017^33^
Chile	Yes^34^	Yes^35^	Yes^35^	PrEP is indicated for someone who is a member of a key population and fulfills the inclusion criteria. Key populations: MSM, trans people, sex workers, those with high HIV risk. Inclusion criteria: a beneficiary of the public health coverage who does not have HIV and is (a) 18 and older, not suspected to have an acute HIV infection, and has had a sexual partner in the past 6 months who has HIV and is not on ART or is not virally suppressed on ART OR (b) is sexually active and presents one of the following in the past 6 months: condomless anal sex with more than 1 partner, or history of STIs, or a sexual partner is one or more HIV risk factors, or uses recreational drugs during sex.^36^	Yes^37^	Available at the national level through the public health system since 2019^36^,^37^
Colombia	Yes^38,39^	Yes^39^	Yes^39^	PrEP is recommended for anyone over 12 years of age with substantial HIV risk. Those who have presented at a recruitment site with one of the following in the past 6 months: history of an acute STI, history of transactional sex, having condomless sex with someone of unknown or positive HIV status, having received non-occupational PEP. However, before initiating PrEP, the patient’s HIV risk should be evaluated on an individual level. Key populations: MSM, transgender people, sex workers, people who consume psychoactive substances, PWID^39^	Yes^40^	Required to be covered for indicated patients through the public insurance program^40^
Ecuador	Yes^41^	Yes^10^	–	For MSM and TW: HIV-positive sexual partner, recent bacterial STI, high number of sexual partners, clinical history that is unclear on the use of condoms, and/or sex work. For heterosexual men and women: HIV-positive sexual partner, recent bacterial STI, high number of sexual partners, clinical history that is unclear on the use of condoms, sex work, and/or high number of HIV in their surroundings. PWID: HIV-positive sexual partner, and/or sharing of injection equipment^41^	No^42^	Available through a demonstration project in cooperation with the Ministry of Public Health and Corporación Kimirina NGO in Quito and Guayaquil^42^
Guyana	Yes^10,43^	Yes^10,43^	–	–	Yes^43^	Available free of cost in certain public health centers and NGOs through a public–private partnership with the Ministry of Health^43^
Paraguay	Yes^44^	Yes^45^	Yes^45^	Eligibility criteria: HIV-negative, belong to a key population (MSM, TW, serodiscordant), not suspected to have acute HIV infection, significant risk of HIV infection (condomless anal or vaginal sexual contact with more than one partner in the last 6 months, or recent STI infection confirmed with a laboratory test or symptomatic infection in the last 6 months, or use of PEP in the last 6 months, or condomless sex with a partner who has HIV and is either not on ART or has a detectable viral load), no contraindication for FTC-TDF, expressed desire to use PrEP and willingness to follow up with HIV testing^44^	No^46^	Available through pilot project at a hospital in the country’s capital^46^
Peru	Yes^47^	Yes^48^	Yes^48^	–	No	Available for free through demonstration projects and clinical research trials, and at cost in certain pharmacies^49^
Suriname	Yes^50^	Yes^10^	–	–	No^50^	PrEP is available through private health centers^50^
Uruguay	Yes^51^	Yes^52^	Yes^52^	For MSM and TW: condomless anal sex with more than one partner in the last 6 months, and/or recurring episodes of STIs, and/or repeated need for PEP, condomless sexual practices with multiple partners in the context of drugs or alcohol, and/or sex in exchange for money or drugs, and/or serodiscordant partner without viral suppression or unknown viral load. For heterosexual men and women: condomless sex with persons at substantial risk for HIV infection and whose HIV status is unknown or is positive without viral suppression, and/or recurring episodes of STIs, and/or repeated need for PEP, and/or sex in exchange for money or drugs^51^	No^53^	Available for pregnant women in serodiscordant relationships. An anticipated pilot project for other ‘at risk’ groups was in planning in 2019, but it is unclear if it was implemented^53^
Venezuela	–	–	–	–	–	–
Central America and Mexico						
Belize	–	–	–	–	–	–
Costa Rica	Yes^54^	Yes^55^	No^55^	Key populations: MSM, TW, serodiscordant partners, other population with an HIV incidence of > 3/100 person-years. Inclusion criteria: belong to a key population and be 18 year-old or older, not present contraindication for the medication, have a negative HIV test, agree to comply regularly with the recommendations and adhere to a longitudinal program, and, because of their characteristics, they have a significant risk of contracting HIV, such as (a) sexually active person in a group with high HIV incidence/prevalence, (b) persons who live in a geographic region with high prevalence, (c) persons who have, in the past 6 months, presented with a risk behavior like condomless anal and/or vaginal sex with more than one partner, or condomless anal and/or vaginal sex with one partner with unknown or positive HIV status, or sex under the influence of alcohol or other drugs, or remunerated sexual conduct, or history of other STIs determined through laboratory tests or treatment of a symptomatic STI, or a sexual partner with one or more HIV risk factors^54^	Yes^56^	Available at public health clinics through a pilot program for those who have public insurance. Also available at private clinics for cost^56^
El Salvador	Yes^57^	Yes^58^	Yes^58^	PrEP is designated exclusively for people who are HIV-negative that belong to a population group with high HIV risk from the following list: 1. MSM. 2. TW. 3. People who engage in sex work. 4. People who practice transactional sex. 5. People with high HIV risk such as serodiscordant couples, women who are pregnant or nursing (with special considerations), or mobile populations (with special considerations). 6. Adolescents with high HIV risk. 7. Other populations that have unprotected sex with high HIV risk.^57^	Yes^57^	National guidelines published in December 2021 for the implementation, promotion, monitoring, and evaluation of PrEP in the public health system and NGOs that provide HIV care and prevention. It is unclear if these have been implemented and if PrEP is currently available through the public health system.^57^
Guatemala	Yes^59^	Yes^60^	Yes^60^	MSM and TW who have had condomless sex in the past 6 months and at least one of the following: sex with more than 2 partners, diagnosis of one or more STIs, use of PEP, and/or use of psychoactive substances during sex. In addition, PrEP is available to people with an HIV-infected partner without clinical or virologic control and without condom use, people who have non-protected transactional sex, PWID who share syringes, people in socially vulnerable situations who have unprotected sexual exposure with HIV high risk.^59^	No^61^	Available through an NGO-sponsored implementation project^61^
Honduras	In planning^62^	–	–	–	–	–
Mexico	Yes^63^	Yes^10^	No^10^	Basic inclusion criteria: (a) negative test for HIV, (b) belongs to a key population, (c) high HIV exposure, and (d) does not present with contraindications for PrEP. Key populations: MSM with high exposure to HIV, transwomen, adolescents with high exposure to HIV, women and men with a serodiscordant partner without virologic control, psychoactive substance users with high-risk practices, sex workers, and PWID^63^	Yes^64^	Available through demonstration projects. As of December 2020, PrEP has been offered through the public health system in a pilot program for certain regions of the country.^64^
Nicaragua	–	–	–	–	–	–
Panama	Yes^10^	Yes^65^	Yes^65^	–	Yes^66^	Available at the national level through the public health system since 2022^66^
Caribbean						
Antigua and Barbuda	No^67^	No^67^	No^67^	–	–	–
Bahamas	Yes^10^	Yes^10^	Yes^10^	Those in a relationship with an HIV-positive partner, persons who have unprotected sex with sex workers, men who don’t use condoms when having sex with men, persons who have been diagnosed with an STI in the past 6 months and who are not in a mutually monogamous relationship with an HIV-negative partner^68^	Yes^10^	Available at the national level through the public health system since 2018^10^
Barbados	Yes^10^	Yes^10^	Yes^10^	Adult person (>18 years old) who is also HIV-negative with no suspicion of acute HIV infection and at least one of the following in the past 6 months: 1. Is in an ongoing sexual relationship with an HIV-positive partner who is not virally suppressed; 2. Is a man who has sex with men (MSM) engaging in unprotected anal sex with another man (receptive or insertive); 3. Is a transgender individual engaging in unprotected sex (vaginal or anal); 4. Exchanges sex for money or goods and engages in unprotected sex (vaginal or anal); 5. Is a MSM, transgender individual or a person that exchanges sex for money or goods with diagnosed or reported STI; 6. Has unprotected sex (vaginal or anal) with 1 or more partners of unknown HIV status who are known, or believed to be at substantial risk of HIV infection; and 7. Had PEP for sexual exposure.^69^	Yes^10^	Available at the national level through the public health system since 2018^10^
Cuba	Yes^70^	Yes^10^	Yes^10^	–	Yes^10^	Available through a pilot project for 28 people beginning in 2019^71^
Dominica	No^6^	–	–	–		
Dominican Republic	Yes^72^	Yes^73^	Yes^73^	Priority populations for PrEP use: MSM, TW, male and female sex workers, migrants, serodiscordant couples, PWID. Eligibility requirements: 13 years of age or older, negative for HIV, have an HIV-positive partner, have had a recent (in the last 6 months) bacterial STI, have a high number of sexual partners (⩾2 different partners in the past 6 months), history of condomless sex or inconsistent use of condoms (during insertive or receptive vaginal or anal sex), participates in commercial sex work, consumes psychoactive drugs, shares drug injection equipment^72^	No^10,50^	Available through an implementation project and private health centers^10,50^
Grenada	–	–	–	–	–	–
Haiti	Yes^74^	Yes^75^	Yes^75^	PrEP is reserved for confirmed HIV-negative people who consent to PrEP and are part of a key population: MSM, Sex workers, PWID, transgender people, or those who are in a serodiscordant couple.^74^	Yes^76^	Available at the national level through the public health system since 2019^76^
Jamaica	Yes^10^	Yes^10^	–	–	No^10,50^	Available through a demonstration project and private health centers^10,50^
St. Kitts and Nevis	–	–	–	–	–	–
St. Lucia	–	–	–	–	–	–
St. Vincent and the Grenadines	–	–	–	–	–	–
Trinidad and Tobago	–	–	–	–	–	–

(–), not available; ART, antiretroviral treatment; HIV, human immunodeficiency virus; MSM, men who have sex with men; NGO, non-governmental organization; PEP, post-exposure prophylaxis; PrEP, pre-exposure prophylaxis; PWID, people who inject drugs; STI, sexually transmitted infection; TDF-FTC, tenofovir disoproxil fumarate/emtricitabine; TW, transgender women.

Since its adoption by the WHO in 2014, oral PrEP had been the only globally approved route of medication delivery until 2021, when the US Food and Drug Administration (FDA) approved cabotegravir, a long-acting injectable form of PrEP.^77^ To date, no country in Latin America has approved cabotegravir for use, and Brazil is the only country currently reviewing it for approval.^78^ Although cabotegravir is not available through the public health system or for purchase at pharmacies in Latin America, cabotegravir and other PrEP modalities are available through randomized controlled clinical trials and subsequent open-label extension studies in Peru, Brazil, and Argentina.^79^

### Unknown status of PrEP approvals

Our findings show a gap of information about policy-level PrEP approvals in many LAC countries. Within country-level Ministry of Health pharmaceutical registries, it is often difficult to distinguish between approvals for branded Truvada and generic TDF-FTC, which is important as countries use generic medications to reduce the cost of PrEP. In addition, sources at times did not distinguish whether Truvada or TDF-FTC is approved for PrEP or only as HIV treatment. For example, Bolivia lists an approval for branded Truvada on their Health Registry of National and Imported Medications, but there are no other references to Truvada on the Ministry of Health website. Although the medication is approved, there is a lack of information on its availability, indications for use, usage, and future policy plans in Bolivia.^31^

Findings document a lag between the media and gray-literature announcement of PrEP availability and implemented country-level policies. In Guyana, a January 2021 article from UNAIDS announced that ‘Guyana will roll out a comprehensive plan for pre-exposure prophylaxis’.^43^ A local Guyanese newspaper published an article in 2021 stating PrEP is currently available in certain sites through the public health system. We were, however, unable to find documents from Guyana’s Ministry of Health outlining their PrEP policies. This lack of policy documents from Guyana may be due to PrEP being approved in a country before national policies or clinical guidelines are written and published.

This dearth of country-level PrEP-related documentation is reflected in other sources that report on PrEP policies. ‘PrEP Watch’ and AVAC provide centralized information on global PrEP availability. The PrEP Watch Global Tracking page shows no PrEP approvals for Colombia, Costa Rica, El Salvador, Suriname, Uruguay, and Paraguay even though our review found country-level documents for PrEP approvals in those LAC countries. Within PrEP Watch and AVAC reports, there is a lack of information on Latin American PrEP policies compared with PrEP in the Global North and Sub-Saharan Africa.

### PrEP implementation and cost

Most countries (14/22; 64%) where PrEP is approved also had clinical guidelines for PrEP published by the country’s Ministry of Health. All available guidelines were published between 2018 and 2022. All country guidelines also outlined specific key populations who may benefit from PrEP use, including MSM, sex workers, and serodiscordant couples. In addition, nine of these countries specifically included TW in their guidelines (Argentina, Costa Rica, the Dominican Republic, Ecuador, El Salvador, Guatemala, Mexico, Paraguay, and Uruguay). The other five countries (Barbados, Brazil, Chile, Colombia, and Haiti) named ‘transgender individuals’ or ‘transgender persons’ instead of transwomen as a key population. Finally, eight country guidelines named people who inject drugs (PWID) as a key population (Argentina, Colombia, the Dominican Republic, El Salvador, Ecuador, Guatemala, Haiti, and Mexico).

Beyond key populations for PrEP provision, our analysis shows a heterogeneous landscape of indications and ‘risk behaviors’ in national PrEP guidelines. Most countries included inconsistent condom use during sex or condomless anal sex with multiple partners or a history of sexually transmitted infections (STIs) as inclusion criteria for key PrEP populations. There were, however, differences in phrasing that allowed for discretion between the patient and the provider. For example, Ecuador listed specific, clear criteria that define ‘substantial risk’ for HIV infection based on population groups.^41^ Other guidelines were less rigid and more flexible. Barbados’s clinical guidelines included the following note on eligibility criteria:

Other individuals who may not fit within the above risk categories may qualify for PrEP or may be requesting PrEP based on perceived risk of exposure. Decisions to initiate PrEP should be individualized by weighing patients’ personal risk of acquiring HIV infection against the potential benefits and risks of TDF-FTC.^69^

These guidelines allow the individual at perceived risk for HIV to discuss the potential use of PrEP with their provider even if they do not belong to a key population but are at risk of HIV exposure. In addition, Chile, Colombia, Costa Rica, El Salvador, and Guatemala had similarly open language that allow for a more individualized assessment of the potential benefits of PrEP for patients who may live outside of changing identity labels.

Of the 22 countries that have approved oral PrEP, 13 have incorporated PrEP into their public health system, while 9 have yet to do so. Countries that offer PrEP as a public policy generally provide it through government-run public health clinics. Health centers that provide PrEP must meet specific infrastructure and personnel requirements that are outlined in each country’s PrEP guidelines (Table 1). In 2017, Brazil became the first country in the region to provide free PrEP through its public health system to key populations.^33^

There are varied modes of PrEP availability for countries that have approved PrEP, but do not offer it as a public health policy. Certain countries (i.e. Costa Rica, the Dominican Republic, Jamaica, Peru, and Suriname) have PrEP available for purchase at commercial pharmacies and private clinics. In other countries (Argentina, Brazil, Peru^79^), PrEP can be available within public systems and to participants in research studies. This included participants in studies evaluating novel PrEP technology, like the HIV Prevention Trials Network (HPTN 083) study evaluating long-acting, injectable PrEP.^79^ Participants in such countries could also access PrEP in demonstration and implementation studies that evaluate the efficacy of PrEP scale-up such as the ImPrEP study in Brazil, Mexico, and Peru, from 2018 to 2021.^80^ Peru, for example, has non-governmental organization (NGO)-run implementation projects that provide PrEP medication for free but may not cover the cost of the associated laboratory monitoring and STI testing. There are also HIV Vaccine Trials Network (HVTN) and HPTN sponsored trials that are required to offer PrEP to trial participants.^49,81^

In countries where PrEP is approved, the cost of PrEP is often unclear. Across LAC, there is limited information on the cost of PrEP in each nation. Out of the 22 countries where PrEP is approved, only Ecuador published the cost of PrEP in its national health ministry guidelines. The price limit for TDF-FTC set by the Ecuadorian Ministry of Health is 4.57 USD/month.^82^ News media occasionally reports on the cost of PrEP in a country; however, there is often a great amount of disparity across different reports, leading to a lack of clarity as to the cost of PrEP in the region. For example, in Chile, one article from 2018 cites PrEP as costing between 70 and 105 USD per month for Truvada, and 42 USD per month for generic TDF-FTC.^83^ However, an article from 2019 reports the cost as being significantly higher, between 575 and 645 USD per month.^84^

The largest regional initiative to lower the cost of PrEP is the PAHO Strategic Fund. Our review of the fund showed 31 governments have signed on to the agreement, including three territories and 31 countries. Although 34 countries and territories in the Latin American region are members of the PAHO Strategic Fund, there is an absence of information to discern which are using the fund to procure PrEP at a reduced rate, as each request the medications they wish to access from PAHO. With little public information on the types of medications purchased through this agreement, it is difficult to assess the success of this initiative specifically with regard to making PrEP more accessible.

## Discussion

This scoping review synthesized the status of country-level PrEP policies across LAC to better understand ongoing PrEP implementation gaps and opportunities. The findings presented here underscore a diverse PrEP policy and access landscape across LAC. The majority, 22 of 33, of LAC countries have approved daily, oral PrEP for use; 15 of these countries have approved the use of generic TDF-FTC, and none have approved injectable cabotegravir. While PrEP is poised to be a significant tool to aid HIV prevention efforts in the region, access remains variable and uncertain as there is a lack of published information on PrEP approvals, policies, guidelines, and cost in many countries throughout the region. This review illustrates that the present situation of PrEP policies in Latin America lags compared with policies in many countries in the global North and sub-Saharan Africa. There is therefore a need to ensure that the ongoing and future rollout of long-acting and other PrEP modalities is equitable globally while also ensuring the continued rollout of oral PrEP.^15^

Findings presented underscore various important facets to consider regarding implementation of PrEP policies in the region. First, PrEP, using either a branded or generic formulation, must be approved for prevention by the country’s government. Eleven countries in LAC have not approved PrEP or have not published data on PrEP approvals in their country. The incorporation of PrEP into a combination HIV prevention approach for key populations in these 11 countries could provide an opportunity to reduce HIV infections.

Second, national clinical guidelines for PrEP must be issued to health care providers, outlining key populations and inclusion criteria. Yet, there are differences among definitions for key populations and their respective inclusion criteria. Recognizing categorizations of key populations and language to describe them change over time, it may be helpful to see inclusion criteria that allow for inclusion and flexibility.^85^ Much of the literature and PrEP clinical guidelines refer to MSM and TW as key populations within the LGBTQ+ (lesbian, gay, bisexual, transgender, and queer/questioning) category who may benefit from PrEP.^11^ There are, however, others who do not fit those definitions who may be at risk for HIV exposure. For example, non-binary people who have sex with MSM and TW or cisgender men who have sex with TW do not fall neatly into the boxes of ‘men who have sex with men’ and ‘transgender women’. This linguistic distinction underscores the importance of less restrictive clinical guidelines that allow for the patient and provider to make decisions around PrEP use at the individual level. Inclusive and evolving language in country-level PrEP guidelines is an opportunity for governments to address potential gaps in PrEP provision. Clinical guidelines establishing specific key populations and inclusion criteria are important when establishing PrEP policies, as recent literature suggests the cost-effectiveness of PrEP programs depends on their ability to integrate into the existing health system and create appropriate demand from key populations.^86^

Another key population included in clinical guidelines are PWID, with 8 of the 14 countries mentioning PWID in their guidelines. Although the paucity of research on the efficacy of PrEP in PWID may contribute to government hesitancy to include PWID in PrEP policies, there is significant international literature justifying the inclusion of PWID as key population for PrEP use.^87,88^ This is an important opportunity for countries to include PWID in their national guidelines, especially for those who have yet to draft guidelines.

In addition to variations among key populations included in clinical guidelines, there were variations in recommended PrEP modalities as well. ED-PrEP has been shown to be highly effective in MSM and has been recommended by the WHO and holds promise as an alternative PrEP modality for MSM who believe their frequency of risk-contacts do not warrant daily, oral PrEP use. This review revealed seven country-level guidelines that recommend the use of ED-PrEP, although it is important to note that most of the country guidelines that do not mention ED-PrEP were published before 2019, when it was first recommended by the WHO. Countries that revise or draft new PrEP guidelines should include ED-PrEP in their recommendations to provide another prevention modality for MSM with infrequent high-risk sexual behavior.

In LAC, 13 countries have adopted PrEP as a public policy and provided PrEP for key populations, starting with Brazil in 2017. Brazil’s participation in the iPrEx trial from 2007 to 2010 and its own PrEP demonstration project from 2014 to 2016 paved the way for Brazil to provide PrEP through the public health system.^89–91^ More recently, the ImPrEP demonstration project investigated same-day PrEP initiation and adherence in Mexico, Peru, and Brazil.^18^ Results from ImPrEP demonstrate ‘same-day initiation was feasible and safe with good levels of early continuation and adherence’, overall.^18^ These results along with the recent adoption of PrEP as a public policy by a number of LAC countries show PrEP implementation may be feasible in other LAC countries.^92^ Recognizing that LAC is not a monolith, there may be other barriers associated with increased PrEP policies in lower income countries. For example, the ImPrEP project was conducted in three upper-middle income countries.^93^ More research on the feasibility and cost-effectiveness of PrEP is needed in lower-middle-income countries in LAC.

This review highlights the active progress in the region to incorporate PrEP within health systems and, accordingly, LAC countries are in different stages of PrEP approval, guideline development, and availability. Of the 22 countries that have approved PrEP, only 13 countries have made PrEP available through the public health system for key populations, with Panama being the most recent country to adopt PrEP into their system in 2022.^66^ Complicating our assessment were not only ongoing efforts to approve PrEP but also the added dimensions of where approvals are for Truvada or its generic formations *and* approvals from prevention, treatment, or both. This lack of clarity underscores the need for increased transparency and more publicly available information at the national level.

Other countries are on their way toward greater PrEP coverage. Peru, for example, exists in a liminal PrEP policy space where Truvada has been approved for PrEP since 2016 but is not yet provided through the Peruvian national health system.^47^ Furthermore, Peru is currently drafting national clinical guidelines and has several demonstration projects underway or recently completed to determine the optimal framework for PrEP delivery and monitoring.^91^ Clinical guidelines and policies are important to PrEP uptake, as a recent study by Ravasi *et al.*^2^ reported that, among Peruvian providers, their willingness to prescribe PrEP would increase if national guidelines were issued. Decreasing the cost of PrEP, through the PAHO agreement or by using generic medications, may be the biggest opportunity in expanding access to PrEP in the region. As most countries are part of the PAHO Strategic Fund, they could use the fund to order TDF-FTC at a lower cost. To order generic PrEP through the Fund, countries must have approved generic TDF-FTC for use, which three countries that have approved brand name Truvada for PrEP have yet to do. Bridging this gap presents a significant opportunity to lower the cost barrier for PrEP and ensure that PrEP implementation is cost-effective.^94^ To address these barriers to wider PrEP policy implementation in LAC, a list of recommendations is provided in Table 2.

**Table 2.** Suggested recommendations to improve PrEP policies and access in LAC.

National-level recommendations	
1	Countries can approve generic TDF-FTC for PrEP, not just as part of an HIV treatment
2	Countries can sign onto the PAHO Strategic Fund and use the fund to purchase generic TDF-FTC at a reduced cost
3	Countries can create national guidelines for PrEP provision including specific key populations that are most affected by HIV in their country
4	Countries can increase transparency by ensuring online availability of prescription medication approvals and public health policies and updates regarding PrEP
Structural-level recommendations	
1	PAHO, UNAIDS, and other international organizations can work toward establishing PrEP-related indicators for country reporting systems
2	Strengthening regional or global alliances can establish common policies on PrEP access, best practices for medication distribution and monitoring, and ‘buyer’s group’ alliances to obtain favorable pricing or produce generic medications

HIV, human immunodeficiency virus; LAC, Latin America and the Caribbean; PAHO, Pan American Health Organization; PrEP, pre-exposure prophylaxis; TDF-FTC, tenofovir disoproxil fumarate/emtricitabine; TW, transgender women; UNAIDS, Joint United Nations Program on HIV/AIDS.

Key limitations need to be considered when interpreting these findings. Most importantly, this scoping review provides a static snapshot of approved PrEP policies, and, thus, may not incorporate active processes that are ongoing and leading to policy approval. Second, there is a lack of available published information about PrEP policies in LAC as well as a lag between government action surrounding PrEP and available gray literature on the topic. While AVAC’s Global PrEP Tracker, UNAIDS, and PAHO are the leading international organizations reporting on PrEP and HIV prevention, this review found that PrEP approvals and policies from LAC often do not appear in publications by the Global PrEP Tracker, UNAIDS, or PAHO. This absence demonstrates a need for increased transparency of PrEP-related information and public access to information regarding programming and purchasing. Transparency is a powerful framework in global health that can be used to solve issues of corruption, medication procurement, and other problems.^95,96^

## Conclusion

PrEP is a highly effective biomedical HIV prevention tool; yet, to fortify its potential impact, efforts are needed to learn from existing country-level policies to ensure PrEP access across the LAC region. Indeed, PrEP may be particularly suited to aid in combination HIV prevention in LAC, given the concentrated epidemic in the region. While many countries in LAC have approved PrEP for use and have outlined key populations and inclusion criteria for PrEP provision, efforts are needed to increase access to PrEP for key populations. Since 2017, an increasing number of countries in the region have begun to provide PrEP at no cost to key populations, which works to dismantle cost-related barriers for individuals. There are PrEP policy gaps in LAC that need to be addressed including transparency of costing information, increase approvals of generic and long-acting, injectable PrEP, and further comprehensive integration of PrEP in public health systems. Access to PrEP is not just an issue of social justice, but an important public health good that can be cost-effective when using generic medications implemented among the most vulnerable populations.

## Declarations

**Ethics approval and consent to participate:** Not applicable.

**Consent for publication:** Not applicable.

**Availability of data and materials:** The data that support the findings of this study are available from the corresponding author, AP-B, upon reasonable request.
